# A Novel Mechanism for Autoantigenicity: Condensate Conformational Change

**DOI:** 10.3390/biom16060803

**Published:** 2026-05-29

**Authors:** Howard M. Fried, Philip L. Carl

**Affiliations:** 1 Department of Biochemistry and Biophysics, University of North Carolina at Chapel Hill, Chapel Hill, NC 27599, USA; 2 Department of Pharmacology, University of North Carolina at Chapel Hill, Chapel Hill, NC 27599, USA; pcarl@bellsouth.net

**Keywords:** autoantigenicity, epitope, B cell recognition, immune system, biomolecular condensate, protein structure, protein–protein interaction, protein dynamics

## Abstract

Autoantigens, targets of B cell antibodies, account for ~10% of the human proteome. It is not well understood why or how particular self-proteins become autoantigens. Prominent theories posit that failures of the immune system at various levels or exposure of aberrant proteins to the immune system are responsible for autoantigenicity. In contrast, we propose a completely unrelated mechanism of autoantigenicity that is based on normal cell functioning. We suggest that, when a protein associates with one of the ~100 types of biomolecular condensates, protein–protein interaction therein causes changes in protein structure or conformation that signal an untolerized state to the immune system. This proposal is derived from the fact that many macromolecules are more concentrated within condensates than in the extra-condensate environment. Thus, the propensity for macromolecular interaction may be greater in condensates compared with the non-condensate environment. In support of this proposal, we present examples of predicted conformational differences, with and without accompanying secondary structure differences, between proteins in known condensate heteromeric complexes and in their free monomer forms. In each case, the differences correlated with predicted B-cell binding, supporting our model. Further, from a compilation of ~1900 autoantigens, we estimate that autoantigens are twice as prevalent in condensates compared with the entire human proteome (Chi squared *p* < 0.0001), further suggesting that a property of condensates, e.g., protein conformational change, may contribute to autoantigenicity.

## 1. Introduction

For several decades, immunologists have been puzzled by the question of how particular proteins are selected to become autoantigens. Attempts to address this puzzle have focused on identifying biochemical and biophysical properties shared by autoantigens [1,2,3]. Plotz [1] first remarked that the repertoire of autoantigens is surprisingly limited. He estimated that there existed but a few hundred different autoantigens. Although the number is probably closer to a few thousand, a few thousand autoantigens is still a small fraction of the approximately 20,000 proteins in the human proteome. Plotz commented that “The repertoire of target autoantigens is a wunderkammer, a collection of curiosities of molecules with no obvious linking principle.” Nevertheless, Plotz suggested a handful of protein features that might influence the selection of autoantigens, e.g., structural properties such as a highly charged surface, repetitive surface elements, the presence of a coiled coil, and bound nucleic acid. Shared properties notwithstanding, the observation of a limited repertoire of autoantigens has remained largely unexplained.

More recently Root-Bernstein and Fairweather [4] and Brooks [5] reviewed theories that attempt to explain development of autoantigens and the origin of autoimmune disease. These theories tend to fall into two groups. One group of theories suggests some mistake by the immune system such that a previously tolerized protein is no longer recognized as self, despite not having a change in structure. Theories in the second group posit changes in structure of a previously tolerized protein.

Root-Bernstein and Fairweather further point out that most explanations of the origin of autoimmunity are unifactorial. That is, the theories assume a single cause, often an environmental factor, and evidence that supports one theory almost invariably supports others. Brooks points out the “overwhelming number of variables from environmental factors, genetics and epigenetics” that can influence autoimmunity. These authors caution us not to expect a universal solution for a process as complicated as the generation of autoantigens. Given this complexity, it is not surprising that more than two decades after Plotz noted the wunderkammer of autoantigens, the factors that might unite the rather limited repertoire of autoantigens are still unknown.

Brooks [5] also noted that “Protein dynamics can lead to exposure of a less accessible epitope or protein–protein interactions can form epitopes only seen in protein complexes.” In support of this view, we present evidence for a potential mechanism of generating novel, untolerized epitopes that arise from changes in protein conformation produced by protein–protein interaction.

The protein–protein interactions we refer to occur within biomolecular condensates (hereafter referred to simply as condensates). There may be as many as 100 different types of condensates in human cells [6]. Condensates conduct a myriad of functions, including steps in gene expression, metabolism, nuclear transport, and signaling [7]. Many types of condensates are produced in response to cellular stress [8], and the composition of a given condensate may vary with other environmental conditions, activities of signaling pathways, and changes in gene expression [9,10].

Condensates are attractive targets for investigating the effects of protein–protein interaction. Condensates are not enclosed in lipid membranes; instead, they are produced by a process known as liquid–liquid phase separation [11,12]. Phase separation selectively concentrates proteins relative to the extra-condensate milieu [13,14]. In addition, as Sabari et al. point out, compared with the entire cell, proteins that form complexes are expected to find each other more easily in condensates because of “smaller search volumes” [15]. Thus, elevated protein concentration and confinement in condensates likely promote protein complex formation. We hypothesize that, if the immune system gains access to protein complexes within condensates (by possible pathways discussed later), the immune system may recognize new, untolerized epitopes arising from changes in protein conformation and mount an antigenic response against one or more proteins in the complexes.

To test this hypothesis, we used bioinformatics (Complex Portal, CD-CODE) and structure prediction tools (AlphaFold, ScanNet) to compare the structures of selected condensate complexes with the structures of the free, monomeric forms of their constituent proteins. Each of the complexes we studied contained at least one reported autoantigen. In each case, we found that predicted conformational differences between proteins in known heteromeric complexes and in their free monomer forms correlated with predicted B-cell binding. It remains to be demonstrated that the new epitopes are actually produced in vivo or recognized by antibodies. Incidentally, in an earlier report [16] we noted many similarities between the biochemical properties of condensate proteins and autoantigens, and we suggested that “high concentration and tendency to form novel complexes with other proteins may partially explain why condensates are potent sources of autoantigens.” At the time, some tools did not exist to assess that idea. In this study, we now address that hypothesis. In sum, we suggest that protein–protein interaction, driven by the environment within condensates, may be a simple, yet overlooked mechanism for the genesis of some autoantigens.

## 2. Materials and Methods

### 2.1. Selection of Heteromeric Protein Complexes

We searched for heterodimeric and heterotrimeric complexes in Complex Portal https://www.ebi.ac.uk/complexportal/home (Build 247, first accessed on 26 July 2024), a manually curated database of experimentally confirmed protein and protein–nucleic acid complexes compiled by EMBL [17,18]. Because the entries in Complex Portal are not annotated with respect to their condensate status, we used key words that were likely to identify biomolecular condensate proteins, e.g., RNA-binding, nucleus, nucleolus, granule, transcription, and translation. Although complexes of greater sizes are abundant in Complex Portal, we limited the search to dimeric and trimeric complexes to simplify subsequent analyses and to accommodate computational limits of various protein analysis services. We used Complex Portal instead of other protein–protein interaction databases because Complex Portal membership requires that complexes “are stable enough in vitro to be reconstituted and have been demonstrated to have a specific molecular function.”

### 2.2. Protein Complexes in Biomolecular Condensates

The individual proteins that comprised the aforesaid complexes were submitted to CD-CODE version 1.08, https://cd-code.org (first accessed on 1 December 2024) to identify complexes whose component proteins are associated with biomolecular condensates. CD-CODE “is a comprehensive, semi-manually curated crowdsourcing database of biomolecular condensates and their constituents” [19]. Currently, CD-CODE version 2.2 lists ~4300 human proteins in >100 human biomolecular condensates.

### 2.3. Autoantigens

An autoantigen collection was assembled from six published studies (Table 1). These studies did not have anything in common with each other, except that four of them were performed using microarrays. Many of the reported autoantigens were not confirmed with citations in PubMed or other databases. Notably, despite the complete independence of each study, 16% of the total reported autoantigens were identified in more than one study, which represents a partial, internal confirmation. In addition, as another partial confirmation, 35% of the autoantigens culled from the six studies was found in the Immune Epitope Database, https://www.iedb.org (first accessed on 14 April 2026) [20]. Thus, we initially selected these studies merely to provide a facile means to identify autoantigens associated with biomolecular condensates.

If necessary, the identifiers for the autoantigens listed with the six reports were converted to UniProt IDs, https://www.uniprot.org/id-mapping (first accessed on 23 August 2024). Some autoantigens were omitted from the final collection for the following reasons: (i) A non-UniProt identifier did not have a corresponding UniProt ID; (ii) A protein was designated as unreviewed in UniProt; and (iii) as indicated earlier, some autoantigens (n = 372) occurred in two, three, or four of the six collections; hence, the duplicate entries were omitted.

### 2.4. AlphaFold Protein Structure Prediction

Structure predictions for the individual free proteins were obtained from the AlphaFold Protein Structure Database, https://alphafold.ebi.ac.uk/ (first accessed on 5 May 2024) [27,28]. The structures of all the protein complexes shown in this report were predicted by AlphaFold 2 Multimer ver 3 (https://neurosnap.ai/), with the AMBER relaxation option to eliminate side chain steric clashes.

### 2.5. Per Residue Secondary Structure

The aforesaid AlphaFold-predicted structures were submitted as pdb files to STRIDE http://stride.helmholtz-munich.de/cgi-bin/stride/stridecgi.py (first accessed on 30 August 2024) [29], “a program to recognize secondary structural elements in proteins from their atomic coordinates” [30]. For each residue in a protein, STRIDE assigns either hydrogen-bonded turn, T, alpha-helix, H, 3_10_ helix, G, β strand extended, E, isolated β bridge, B, or coil (none of the above), C. Strictly speaking, coil is not necessarily “random coli”. The “C” designation represents only the lack of hydrogen-bonded secondary structure. However, in crystallographic or NMR analyses, such regions often lack stable structures. Thus, for this report, we equate C with unstructured/disordered. This approach is further justified because the residues in epitopes composed of all or mostly C (see Section 3) had AlphaFold pLDDT values < 40, values that indicate lack of structure.

### 2.6. Epitope Prediction

The structures of free proteins and protein complexes were submitted as pdb files to ScanNet, https://neurosnap.ai/ (first accessed on 30 August 2024) to predict B-cell epitope binding sites. For each amino acid residue, ScanNet reports a single metric, namely, the probability (0.0–1.0) that a residue is part of a B-cell epitope. A probability of 35% was used as the threshold for B-cell recognition. According to its developer Jerome Tubiana, “ScanNet’s positive training set is incomplete, and many epitopes are missing due to lack of solved antibody–antigen complex. Therefore, the threshold 0.35 was manually determined from a precision-recall curve and by visual inspection of a small test set. A threshold of 0.35 corresponded to high precision (>90%), but moderate recall”.

For protein complexes, the option “Assembly” was selected so that all chains in a complex were analyzed as assembled, instead of being analyzed independently of each other. Residues that had the same binding probabilities in the assembled complex and in the individual (independent) chains in the complex were deemed to be exposed in the complex; that is, their accessibility was not affected by the presence of partner proteins. Conversely, residues that had low binding probabilities when the chains were considered together, but higher binding probabilities when the chains within the complex were analyzed independently, were residues that were not fully accessible in the complex [31].

### 2.7. Correlation of Epitopes with Conformational/Structural Change

A Python (ver. 3) script (by Dr. Keaun Amani, Neurosnap, Inc., Wilmington, DE, USA) was used to align the structures using PyMOL (Version 3.0, Schrödinger, LLC, New York, NY, USA) and to identify all C-α’s whose positions differed by >2 Å between the monomer and complex forms of a protein. Residues meeting this requirement were then examined for both their B-cell epitope predictions from ScanNet and their secondary structures assigned by STRIDE. According to Dr. Amani, “the >2 Å threshold is widely used to identify meaningful Cα displacements. The value is above typical coordinate uncertainty in experimentally determined or predicted structures, and it enables the detection of subtle but potentially functionally relevant differences, instead of restricting analysis to large structural rearrangements”.

### 2.8. Summary of Evaluated Protein Complexes

We obtained 38 autoantigen-containing condensate protein complexes. Twenty-two of these complexes were rejected because of poor AlphaFold 2 Multimer structure metrics (see Section 3.1). Of the remaining sixteen complexes, seven did not show any epitopes specific to the complexes (essentially, negative controls). The final nine complexes form the basis for this study.

### 2.9. Molecular Dynamics Simulation

Molecular dynamics simulation was performed by Dr. Keaun Amani using GROMACS software (version 2024.4) on the Neurosnap computational platform (https://neurosnap.ai/). Please refer to Supplementary File S1 for complete experimental details.

### 2.10. Molecular Visualization

PyMOL Version 3.0 (Schrödinger, LLC, New York, NY, USA) and UCSF ChimeraX [32] were used to visualize protein structures. ChimeraX was developed by the Resource for Biocomputing, Visualization, and Informatics at the University of California, San Francisco, NIH R01-GM129325, and the Office of Cyber Infrastructure and Computational Biology, NIAID.

### 2.11. Comparison of Autoantigen Prevalence

There is little, if any published data on the estimated number of autoantigens (as native proteins) in the human proteome. We suggest that our collection of 1925 autoantigens, 9.6% of the human proteome, is a reasonable estimate. We also found that 953 of the ~4300 human condensate proteins are autoantigens in our collection. Thus, the autoantigen prevalence in the human condensate proteome is 22% (953/4300), roughly a factor of 2 (chi-squared test *p* < 0.0001) greater than the prevalence in the entire proteome.

### 2.12. Generative Artificial Intelligence

Generative artificial intelligence was not used in the preparation of this report.

## 3. Results

### 3.1. Study Design

The purpose of this study was to identify epitopes predicted to arise from conformational changes in heteromeric protein complexes. The complexes of interest, experimentally confirmed in the Complex Portal database, were composed of proteins reported to be associated with biomolecular condensates. We hypothesized that, when proteins form complexes in condensates, new epitopes (or enhanced existing epitopes) would appear in the complexed forms relative to the uncomplexed (monomer) forms of the proteins.

**Figure 1:**
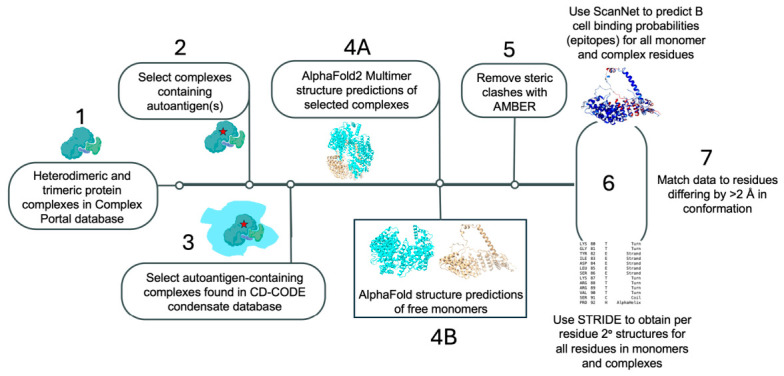
Flow diagram of study: Identifying epitopes predicted to arise from conformational changes in condensate protein complexes. (1) Heterodimeric and heterotrimeric protein complexes (<3000 amino acid residues) were identified in Complex Portal, (https://www.ebi.ac.uk/complexportal/home). (2) The aforesaid complexes were compared with an in-house list of ~1900 autoantigens to select complexes in which at least one protein was an autoantigen (red star). (3) The individual proteins in autoantigen-containing complexes were then submitted to CD-CODE (https://cd-code.org/) to identify complexes for which all members were in the same condensate (large cyan blob). (4A) Autoantigen-containing condensate complexes were submitted to AlphaFold2 Multimer (https://neurosnap.ai/) to predict their structures. (4B) The predicted structures of the individual (free) proteins were downloaded from the AlphaFold Protein Database, (https://alphafold.ebi.ac.uk/). (5) The AlphaFold installation also performed AMBER relaxation to eliminate side chain steric clashes. (6) STRIDE (http://stride.helmholtz-munich.de/cgi-bin/stride/stridecgi.py) was used to obtain the secondary structures of all residues in the monomers and complexes, and ScanNet (https://neurosnap.ai/) was used to predict B-cell binding probabilities (epitopes) for all residues. (7) Lastly, residues with B-cell binding probabilities greater than 35% and greater in the complexes compared with the corresponding monomers were matched with the amino acid residues whose C-α’s differed by >2 Å in conformation (identified with a Python script provided by Neurosnap, Inc.) between monomer and complex forms of the autoantigens. The residues that met these criteria of binding site probability and conformational difference were proposed to be new epitopes in the complexes. Heterotrimer image from BioRender.com.

Figure 1 shows a flow diagram of the study. The first goal was to identify relatively small human protein complexes that contained one or more autoantigens and whose constituent proteins are all associated with the same condensate. Thus, we searched the EMBL Complex Portal [17] for dimeric and trimeric complexes. We then compared the constituents of those complexes with a list of 1925 autoantigens that we compiled independently (see Section 2). Protein complexes that contained at least one autoantigen were then screened with the CD-CODE condensate database [19] to identify autoantigen-containing complexes associated with condensates.

The second goal was to use AlphaFold 2 Multimer [33] to predict structures of the autoantigen-containing condensate complexes. We considered only the top model of the five models returned by AlphaFold for any given complex. In addition, we considered only complexes that had AlphaFold 2 Multimer pTM (“accuracy of overall predicted structure”) scores of ≥0.5 and ipTM (“accuracy of predicted relative positions of subunits”) scores ≥ 0.6 (see Course Contents at https://www.ebi.ac.uk/training/online/courses/alphafold/; first accessed 15 December 2024). In parallel, we downloaded the predictions for the individual (monomer) proteins from the AlphaFold Protein Structure Database.

The third goal was to identify epitopes predicted to be present in the autoantigens within the complexes but absent from the autoantigen monomers. We used ScanNet [31], which predicts on a per residue basis “B cell binding probability,” that is, whether a residue is part of a B-cell epitope. We selected ScanNet because it analyzes structures; many epitope predictors accept only sequences, and we needed to compare two conformations of one protein. Further, ScanNet can analyze multiple chains together “as a single biological assembly” [34]; other epitope predictors accept only single chains. As well, ScanNet can be configured to analyze the chains within a complex independently of each other. This option reveals the relative accessibility of residues (see Methods). ScanNet sometimes predicted epitopes in long unstructured segments. Generally, we did not consider those epitopes because the AlphaFold confidence scores for long unstructured segments are “strong predictor[s] of disorder” (https://alphafold.ebi.ac.uk/faq#faq-12; first accessed 15 December 2024). AlphaFold thereby assigns apparently arbitrary locations to such segments, making a comparison of monomer versus complex conformation untenable.

To identify epitopes present in the autoantigens within the complexes but absent from the free autoantigens, we simply calculated the ratio *R* of the ScanNet B-cell binding probability for each residue in the complex to its binding probability in the monomer. An *R* > 1 indicated that a residue was part of a new (or enhanced) epitope. Because a binding probability of 35% is generally accepted as the ScanNet threshold for B-cell binding (see Section 2.6), we focused on residues that had R values > 1.05 and binding probabilities ≥ 35%.

We also submitted the structures of acceptable complexes and their monomers to STRIDE [30], which reports the secondary structure, and lack thereof, for each amino acid residue in a protein (see Section 2.5 for definitions of secondary structure assignments).

Having identified autoantigens present in condensate protein complexes and potential epitopes in those autoantigens present in the complex but absent from the free monomer form, our last goal was to determine which complex-specific epitopes were associated with conformational change. Thus, we used a Python script (provided by Neurosnap, Inc.) to identify all residues whose C-α positions differed by >2 Å between monomer and complex (see Section 2.7). We reasoned that any new epitope that arose in a complex must be associated with a change in conformation, with or without a change in secondary structure. Without such a conformational change, ScanNet would likely report identical results between monomer and complex forms. We chose a threshold of >2 Å as the smallest meaningful difference, so as to have a baseline to collect the greatest number of conformational differences.

In the following sections, we present examples of epitopes predicted to arise from conformational changes in experimentally confirmed protein complexes. We describe nine human protein complexes, six heterodimers and three heterotrimers, that met the requirements for condensate association, autoantigen presence, and quality AlphaFold structure prediction metrics. The nine complexes represent fourteen autoantigens (only twelve were amenable to study).

### 3.2. Epitopes Arising from Protein–Protein Interaction

In the following Section 3, there are nine Tables containing the data we used to deduce epitopes. For simpler reading, Supplemental File S4 summarizes the salient features of the data.

#### 3.2.1. X-Ray Repair Cross-Complementing Proteins 6 and 5

The XRCC6-XRCC5 (aka Ku70-Ku80) heterodimer (Complex Portal #CPX-1993) mediates DNA non-homologous end joining [35]. According to the CD-CODE condensate database, both proteins are found in the nucleolus and (cytoplasmic) P-body. Both proteins are autoantigens [21,36]. The AlphaFold Multimer structure metrics for the predicted complex were pTM = 0.73 and ipTM = 0.80. Thus, both XRCC6 and XRCC5 met our selection criteria.

##### XRCC6

**Figure 2:**
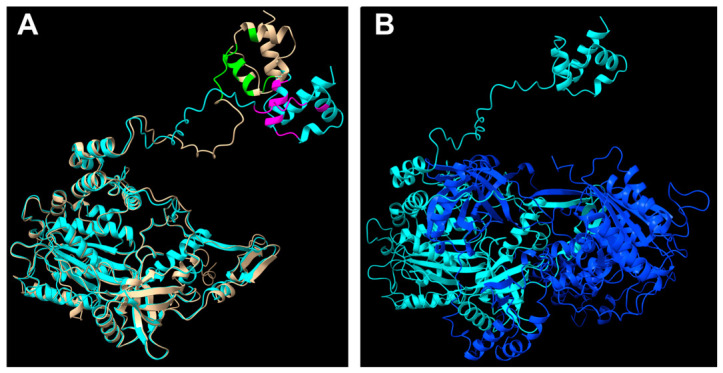
(**A**) Overlay of XRCC6 monomer (sand color) and XRCC6 (cyan) in the XRCC6-XRCC5 complex (XRCC5 not shown for clarity). Green (upper right of image) shows residues in C-terminus of XRCC6 monomer proposed to be new epitopes (in magenta) in the complex (see Table 2). (**B**) XRCC6 (cyan) in complex with XRCC5 (blue). The XRCC6 C-terminus is at upper-right as in (**A**). Image shows that XRCC5 does not block access to the C-terminus of XRCC6.

Of the 609 amino acid residues in XRCC6, 129 (21%) exhibited a >2 Å conformational difference between free monomer and the XRCC6/5 complex. Most of these 129 residues had R values < 1, suggesting that they were not likely to be part of B-cell epitopes. However, beginning at arginine 554 and extending to the end of the protein, many residues had an *R* > 1 and, at least for the complex form, binding probabilities of 35% or more. Table 2 summarizes the data for residues 554–609 that met the criteria. The selected residues do not comprise an uninterrupted segment in the protein primary sequence. However, because the structure of XRCC6 in this region consists of a compact group of three helices (Figure 2A), we thought it possible that the region as a whole contains one or more epitopes. That conjecture agreed with Chou et al. [37] who reported that XRCC6 residues 560–609 include a “major conformational” epitope. Lastly, except for residue 554, STRIDE reported that all of the proposed epitope residues in Table 2 had the same secondary structure in the monomer and complex.

Figure 2A shows an overlay of the XRCC6 monomer and complex form and the locations of the residues listed in Table 2. The residues begin in a six-residue unstructured segment and proceed into a three-helix bundle at the C-terminus (upper-right in the Figure). Most of the residues of interest are within the second of the three helices.

Note that the XRCC6 C-terminal helices are not obscured by another part of XRCC6 or by XRCC5. Instead, the C-terminal helices are tethered by an unstructured lead to the main body of the protein (Figure 2B). Consistent with this exposed structure, ScanNet reported that all the XRCC6 residues from 554 to the end of the protein had the same B-cell binding probabilities in the assembled complex and in the XRCC6 chain within the complex (see Methods for an explanation of this comparison); thus, all these residues were predicted to be exposed in the complex. Lastly, residues 378–482 of XRCC6 mediate the interaction with XRCC5 [38], and, as expected, in the complex nearly all of residues 378–482 had essentially zero B-cell binding probabilities, presumably because of their interaction with XRCC5.

##### XRCC5

Ninety-three percent of the 732 residues of XRCC5 had a >2 Å difference in position between monomer and complex forms. Three groups of residues met the criteria for new epitopes (Table 3), namely, residues 171–177, 242–247, and 725–729.

**Figure 3:**
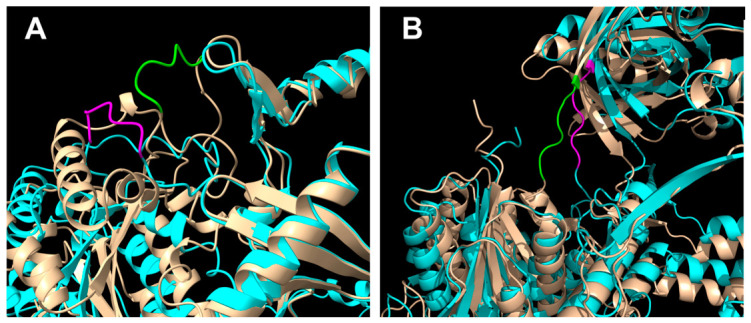
Overlay of portions of XRCC5 as monomer (sand color) and within the XRCC6-XRCC5 complex (cyan). Epitope residues listed in Table 3 are green in monomer; proposed new epitopes in complex are magenta. (**A**) Residues 171–177. (**B**) Residues 242–247. Partner protein XRCC6 not shown to facilitate visualizing essential detail.

XRCC5 monomer residues 171–177 all had ScanNet binding probabilities below the 35% cutoff for B cell recognition, whereas all the residues had favorable probabilities in the complex (residue 177, with a value of 31.9, is included because the threshold is not a precisely determined value). Residues 172–174 transitioned from turn to unstructured (coil in Table 3; Figure 3A). Also, residues 171–177 had the same B-cell binding probabilities in the assembled complex and the individual XRCC5 chain within the complex. Thus, the presence of XRCC6 did not affect accessibility to XRCC5 residues 171–177.

Figure 3B shows another XRCC5 predicted epitope, residues 242–247. Except for residue 242, all the other residues had binding probabilities indicative of a B-cell epitope. Nevertheless, all probabilities were greater in the complex (*R* = 1.05–2.58). Five of the seven residues had slightly lower binding probabilities in the assembled complex compared with the individual XRCC5 chain in the complex, suggesting that their accessibility was slightly obscured. It is important to keep in mind that the structure here is static, and ScanNet considers other features in addition to static accessibility in designating residues as favorable for B cell recognition.

In the monomer, the C-terminal 26 residues of XRCC5 are all unstructured (see green arrow in Supplementary File S2, Figure S1). In the complex, residues 725–729 at the extreme C-terminus all underwent a C-to-T transition (i.e., unstructured-to-structured). As noted in Study Design, in general, we did not consider epitopes predicted in long unstructured segments. However, because of the acquisition of structure, we suggest that residues 725–729 hold promise as an epitope. Note that 725–729 were favorable for B-cell binding despite the segment apparently being positioned in a pocket in the complex (Supplementary File S2, Figure S1). Consistent with this location, residues 725–728 had binding probabilities ~2% lower in the assembled complex compared with the individual XRCC5 chain in the complex.

Lastly, XRCC5 residues 374–502 mediate its interaction with XRCC6 [38]. ScanNet reported that 23 of these residues had B-cell binding probabilities of 12.0–67.0%. However, the binding probabilities of the remaining 106 residues were essentially zero, supporting the earlier identification of the XRCC6 interaction site.

#### 3.2.2. PES1-BOP1-WDR12 Ribosome Assembly Factor

The PeBoW heterotrimer (Complex Portal #CPX-2846) in the nucleolus is required for processing 28S and 5.8S ribosomal RNAs and 60S ribosomal subunit maturation [39]. Metrics for the AlphaFold Multimer structure prediction of the complex were pTM = 0.7 and ipTM = 0.7. PES1 is the autoantigen [22]. Of the 588 PES1 residues, 184 (31%) had a >2 Å difference in conformation between monomer and complex.

**Figure 4:**
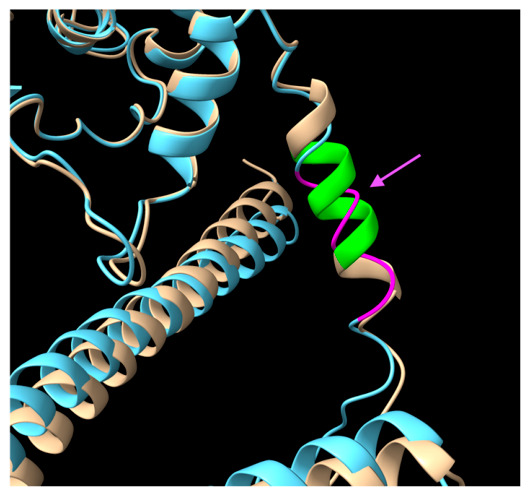
Proposed PES1 epitope. Portion of PES1 monomer is in sand color and complex form is cyan. Green shows residues 455–461 in an α-helix in the monomer. Magenta at same location shows an all-turn structure of 455–461 in the complex (see arrow). Partner proteins BOP1 and WDR12 not shown.

Table 4 shows three suggested new epitopes. Residues 455–461 are helical in the monomer. Intriguingly, in the complex, all seven residues have become turn (Figure 4) with substantial increases in B-cell binding probability. These residues had the same binding probabilities for the assembled complex and the individual chains within the complex, suggesting that partner proteins BOP1 and WDR12 did not conceal the proposed PES1 epitope. Notably, in HSP90, a similar 8-residue helix-to-turn transition is proposed as a new epitope also having a substantial increase in B-cell binding probability (see Supplementary File S3).

Unlike 455–461, residues 60–63 and 108–111 did not undergo changes in structure (except #110 H-to-C), they incurred only small shifts in relative position (Supplementary File S2, Figure S2). However, perhaps the slight relocation may have juxtaposed 60–63 and 108–111 nearer to other residues that somehow enhanced recognition as measured by ScanNet.

#### 3.2.3. STAT1-STAT3 Transcription Activator

Signal transducer and activator of transcription (STAT) complexes transmit the presence of cytokines and growth factors by activating transcription of target response genes [40]. There are seven human STAT proteins. Ligand binding to a cell surface receptor promotes JAK tyrosine kinase phosphorylation of STAT proteins, which then form homodimers and heterodimers and translocate into the nucleus.

We selected the STAT1-STAT3 heterodimer (Complex Portal #CPX-6041) for analysis because CD-CODE describes STAT3 (and by inference STAT1) as associated with the “transcriptional condensate.” STAT1 is the autoantigen [26]. In addition, the AlphaFold Multimer structure metrics for STAT1-STAT3 were pTM = 0.71, ipTM = 0.68, marginally better compared with the metrics for other STAT complexes, e.g., pTM = 0.67, ipTM = 0.59 for STAT1-STAT4 and pTM = 0.58, ipTM = 0.53 for STAT1-STAT2-IRF9.

Of the 750 residues in STAT1, 217 (29%) had a >2 Å difference in conformation between monomer and complex. Four groups of STAT1 residues are proposed as being part of either new or enhanced epitopes in the STAT1-STAT3 complex (Table 5).

In the monomer, a turn created by residues 128 and 129 and followed by helical residues 130–132 join two large α-helices (Supplementary File S2, Figure S3). In the complex, residues 130–132 have become turn. Four of the residues, 128, 129, 131, and 132, contribute to an enhanced epitope (Table 5). Note that the B-cell binding site probabilities for residues 128–132 were identical between the assembled complex and the individual STAT1 chain in the complex, suggesting that some other feature of this segment in the complex contributes to its greater binding probability.

**Figure 5:**
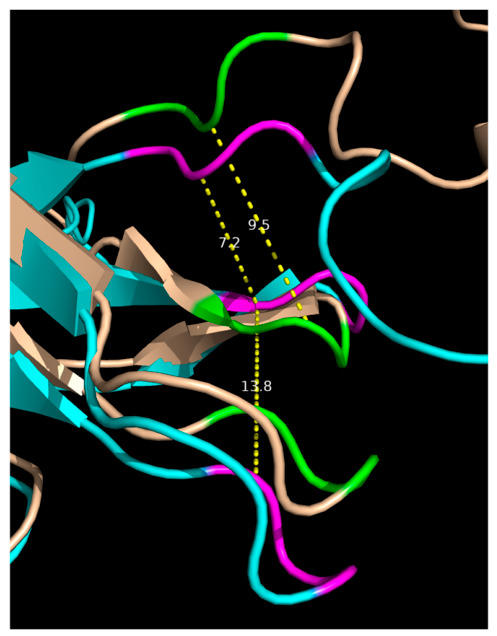
STAT1 epitopes. STAT1 monomer is sand color, and STAT1 in complex with STAT3 is cyan (STAT3 not shown for clarity). Epitope residues (Table 5) are green in monomer and magenta in complex. Top pair = residues 711–713, Middle pair = residues 655–657, Bottom pair = residues 620–622. The dotted lines show distances in Å between the loops. Residues 711–713 and 655–657 are ~9.5 Å apart in the monomer and ~7 Å apart in the complex; their close proximity to each other is suggestive of a discontinuous epitope. Residues 620–622 and 655–657 are ~9 Å apart in monomer and 13.5 Å apart in complex (not shown for clarity).

Residues 620–622 in the monomer and complex are structurally identical, forming a turn. However, the turn in the complex appears to have moved slightly away from the main body of STAT1 (Figure 5). Similarly, residues 655–657 in the complex appear to have moved away slightly from their location in the monomer. Lastly, although residues 711–713 are part of a loop in both forms, in the complex, the loop appears to be closer to the body of the protein. Interestingly, in the complex, the α-carbons of residues 655–657 and 711–713 are ~2.5 Å closer to each other, to within ~7 Å (Figure 5). Thus, the close proximity of the two segments is suggestive of a single B-cell discontinuous epitope (see Discussion; [41]). Note that the binding site probabilities of residues 620–622, 655–657, and 711–713 were the same between the assembled complex and the individual STAT1 chain within the complex, hence, the presence of STAT3 did not hinder accessibility to those STAT1 residues.

#### 3.2.4. ERF1-GSPT1 Translation Release Factor Complex

The ERF1-GSPT1 complex (Complex Portal #CPX-2721) is responsible for terminating translation and releasing the nascent chain at stop codons [42]. Both proteins are found in the Stress Granule condensate. Both proteins are autoantigens [22]. The AlphaFold Multimer metrics for the complex were pTM = 0.84 and ipTM = 0.86. Note: we did not analyze GSPT1 in detail because all potential epitope residues were located in a large (72-residue) unstructured N-terminal tail.

Out of 437 residues in ERF1, 362 (83%) showed a >2 Å difference in conformation between monomer and complex forms. Thus, the overall structures of the two forms were substantially different, mostly because of a large shift in position of structures composed of residues ~146–270 (helix bundle at bottom of Supplementary File S2, Figure S4). None of these residues had B-cell binding probabilities indicative of an epitope. Instead, a handful of residues elsewhere in ERF1 showed B-cell binding probabilities suggesting new epitopes (Table 6).

**Figure 6:**
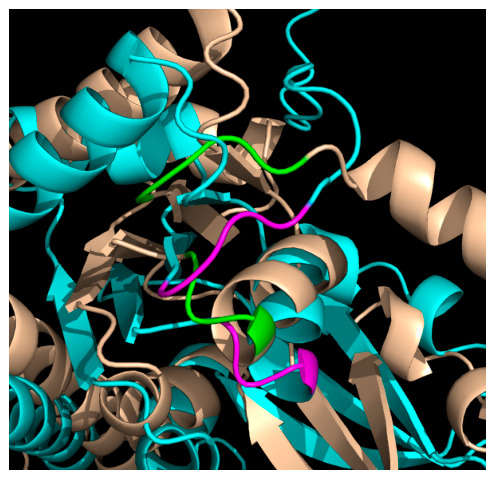
ERF1 epitopes. Epitope residues 314–316 (middle) and 414, 415, and 417 (top); green is monomer, magenta is complex. Note the close proximity of the two groups, suggestive of a discontinuous epitope. Binding partner GSPT1 not shown for clarity.

Residues 369–371 are part of a β-strand; the conformational difference between monomer and complex is small (Supplementary File S2, Figure S4). Residues 314–316 and 414–417 show larger differences in position between monomer and complex. In addition, the two groups of residues appear near each other in space (Figure 6), an average of ~7 Å apart in both monomer and complex, suggesting that together the two groups of residues may be another example of a B-cell discontinuous epitope.

#### 3.2.5. FACT Complex SSRP1 and SPT16 (SUPT16H)

The FACT complex (Complex Portal #CPX-419) destabilizes histone H2A-H2B dimers, enabling RNAPol II passage, after which FACT restores the nucleosomal structure [43]. CD-CODE reports that both proteins are found in P bodies, the Stress Granule, and the nucleolus. Both proteins are autoantigens [21,23,44]. The AlphaFold structure metrics for the predicted complex were pTM = 0.50 and ipTM = 0.61.

##### SSRP1

**Figure 7:**
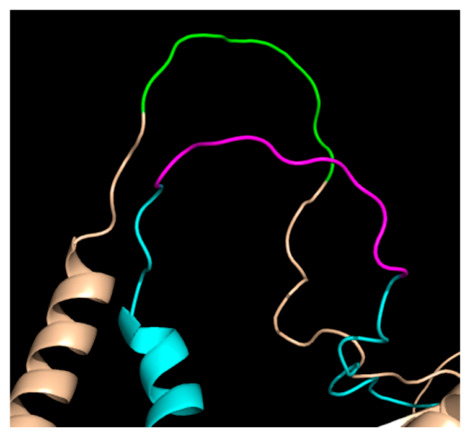
Proposed epitope of FACT complex subunit SSRP1. Residues 437–446 in green are monomer form; magenta shows residues 437–446 in SSRP1-SPT16 complex (SPT16 not shown for clarity). The segment has transitioned from all unstructured (coil) in monomer (sand color) to partial turn in the complex (cyan).

Essentially all of the 709 residues of SSRP1 had >2 Å difference in conformation between monomer and complex forms; most of this difference is attributable to AlphaFold’s apparent arbitrary placement of two ~100-residue unstructured segments. Thus, we found only one new epitope in SSRP1, i.e., residues 437–446 within a relatively short (30-residue) C + T segment. In the monomer, all 10 residues are unstructured. In the complex, the ends of 437–446 have transitioned to turn (Table 7 and Figure 7). The acquisition of structure in the complex suggests that 437–446 may comprise an epitope despite being located in a largely unstructured segment. Note that residues 1–177 of SSRP1 contain the SPT16 interaction domain [43]; thus, SPT16 should not conceal the proposed SSRP1 epitope.

##### SPT16

**Figure 8:**
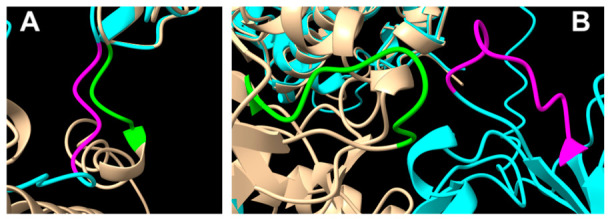
Proposed epitopes of FACT complex subunit SPT16. Portion of free SPT16 monomer is sand color; FACT complex is cyan. Partner protein SSRP1 not shown. (**A**) Epitope residues 432–435; green is monomer, and magenta is complex. (**B**) Epitope residues 846–853 (same color scheme).

Nearly all of the ~1050 residues of SPT16 showed >2 Å differences in conformation between monomer and complex. Indeed, the two forms were barely superimposable. Seven potential new epitopes were found (only two are shown in Table 8 and Figure 8), perhaps a consequence of the shuffling of SPT16’s structure.

SPT16 residues 432–435 were identified by ScanNet as an epitope of the complex (Figure 8A). Residues 431–606 of SPT16 interact with SSRP1. Perhaps the fact that 432–435 are at the end of the SSRP1 interaction site, and all four residues are unstructured (note the change from G helix in monomer to C in complex), enables those residues to be accessed. None of the other six potential SPT16 epitopes mapped to the protein’s SSRP1 binding site.

Most of the other SPT16 monomer–complex pairs were greatly separated in the protein structure, by as much as 40 Å. Figure 8B shows a pair, 846–853, that are relatively close to each other. The two forms appear to be in different environments, which may explain the generation of the epitope.

#### 3.2.6. BUD23-TRMT112 rRNA Methyltransferase

BUD23 23. is a methyltransferase that, along with adapter TRMT112, (Complex Portal #CPX-2871) methylates the N_7_ position of a guanine in 20S pre-rRNA [45]. Both proteins are found in the nucleolus. The AlphaFold Multimer pTM and ipTM metrics were 0.82 and 0.94. BUD23 is the autoantigen [23]. All of the BUD23 residues displayed >2 Å conformational differences between monomer and complex. Half of the differences were small; the remainder were due to an ~100-residue mostly unstructured C-terminus.

**Figure 9:**
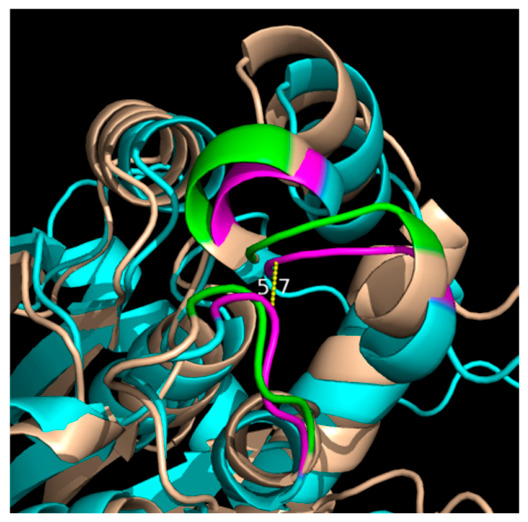
Proposed new epitopes in BUD23. Residues 11, 12, 13, 15, and 16 are at the top, colored in green for free monomer and magenta for BUD23 in complex with TRMT12 (TRMT12 not shown for clarity). Below at the middle are residues 130–133 (same color scheme). Note that the C-α backbone of the two proposed epitopes in the complex are within 6 Å of each other.

Residues 11, 12, 13, 15, and 16 appeared to be an enhanced epitope in the BUD23-TRMT112 complex; four of the five residues hover around binding site probabilities of 35% in the monomer, whereas all of the residues have greater binding site probabilities in the complex (Table 9). Residues 130–133 showed a similar pattern suggesting a second new epitope (Figure 9). Both sets of residues are on the back side of the complex and fully accessible; except for a 10% decrease in the binding probability of residue 130 in the complex, all the residues had the same probabilities in the assembled and individual chains, indicating that the TRMT112 partner did not block access. Note that the C-α backbone of the two proposed epitopes are within 6 Å of each other (Figure 9), suggesting that they may comprise a single discontinuous epitope.

#### 3.2.7. Additional Complexes

Three additional complexes are described in Supplementary File S3. A brief summary is presented here.

A. HSP90-CDC37: This complex (Complex Portal #CPX-3288) is a trimer composed of two HSP90 molecules and one CDC37 molecule. The complex is a molecular chaperone responsible for activating and stabilizing a great many substrate proteins. Both HSP90 and CDC37 are autoantigens, although only one new epitope was found in the complex, namely an 8-residue helix-to-turn transition in both HSP90 molecules. The transition resulted in a substantial increase in binding probability (*R* = ~4–8).

B. KPNA2-KPNB1 nuclear transport complex: The KPNA2-KPNB1 (aka importin-α/β and karyopherin-α/β) heterodimer (Complex Portal #CPX-1027) ferries proteins from the cytoplasm into the nucleus [46]. Both subunits are autoantigens. KPNA2 binds to KPNB1 by way of its 41-residue N-terminal importin-β binding domain (IBB). The IBB appears to be largely surrounded by KPNB1, yet ScanNet predicted that a 5-residue segment of the IBB had substantially greater B-cell binding probability in the complex.

C. EIF2 translation initiation factor complex: The EIF2S1α subunit of this heterotrimer (Complex Portal #CPX-2716) is an autoantigen. Despite an 84% difference in conformation between monomer and complex forms, only one potential new epitope was found, a 3-residue β-strand segment.

#### 3.2.8. Molecular Dynamics Simulation

The proposed new epitopes we describe were based on structures predicted by AlphaFold Multimer and on B-cell binding probabilities predicted by ScanNet. We sought to support our approach with an independent computational analysis, i.e., molecular dynamics simulation. Thus, we selected the XRCC6-XRCC5 complex for simulation. During the simulation, the complex appeared stable because the two proteins did not dissociate, and all simulation metrics indicated stability (see Supplement File S1). In addition, there was limited flexibility across most of the protein complex, with the exception of residues 540–609 of XRCC6 and residues 550–586 of XRCC5. Fluctuations in these regions were consistent with ScanNet’s report that these regions were accessible and that the assignments of favorable B-cell binding probabilities to residues in these regions was appropriate (Table 2 and Table 3). These simulation findings were consistent with the well-known experimentally determined interaction between XRCC6 and XRCC5, and the simulation results reinforced the predictions for XRCC6-XRCC5. Although we simulated only one complex, we suggest that the results validated our study design.

Simulation results, including detailed trajectory and analysis data, are available at https://neurosnap.ai/job/6791957b99464b271efcaf1d?share=67a535fecb419ef7b4982b42 (accessed on 30 March 2025).

#### 3.2.9. Summary of the Results

From the 12 proteins discussed, we propose ~30 groups of residues as representing either new or enhanced B-cell binding sites in heteromeric complexes. We were interested to determine whether any biochemical features were prevalent among the 30 sites. There are many biochemical features that can be assessed, but notably, two-thirds of the binding sites are composed of all, or nearly all, coil and/or turn, and most (80%) of the sites have a net charge at pH 7. In the Discussion that follows, we describe the possible significance of these similarities.

Our study necessitated compiling a dataset of autoantigens. This collection afforded an opportunity to estimate the prevalence of autoantigens among condensate proteins. On the basis of our dataset, ~20% of the condensate proteome is composed of autoantigens, a prevalence that is approximately twice the autoantigen prevalence in the entire human proteome (see Section 4). This experimental finding supports the idea that condensates contribute to autoantigenicity. Notably, we have not confirmed that any of the proposed 30 new or enhanced epitopes arise in cells or are recognized by antibodies.

## 4. Discussion

Proteins in complexes often have local differences in secondary and/or tertiary structure relative to their uncomplexed forms. Thus, the purpose of this study was to predict whether such differences would be recognized as potential B-cell epitopes that are not recognized in the free forms of proteins. Such “new” epitopes would be candidates for untolerized targets of the immune system. The key finding of our study is that some predicted conformational changes correlated with predicted new or enhanced B-cell binding sites, a finding that supports our hypothesis.

To explain autoantigenicity, investigators have sought to deduce shared biochemical features of self-proteins that are targeted by the immune system (e.g., [1,2,3]). As mentioned earlier, Brooks (5) posited that protein dynamics and protein–protein interaction can reveal epitopes. Indeed, antigen structure is highly important in recognition by antibodies. For example, autoantibodies that bind proteins having their proper three-dimensional structures in, e.g., immunoprecipitation assays, often bind poorly to unstructured forms of the same proteins in, e.g., immunoblotting [47,48]. Yet, despite the importance of antigen structure, many studies to identify properties of self-proteins that initiate autoantigenicity have not considered an important state of proteins, namely, heteromeric complexes. Interestingly, Rosen and Casciola-Rosen [48] noted that many highly prevalent autoantigens, e.g., Sm, SRP, RNA Pol I, are components of macromolecular complexes, and often multiple different components of a complex are targets of the immune system.

The proteins we evaluated were associated with one or more biomolecular condensates as contained in the CD-CODE database; their corresponding heteromeric complexes that exhibited new predicted epitopes were from the Complex Portal database of experimentally verified complexes. There are dozens of different condensates with dozens of functions. But one property shared by condensates is concentrating proteins relative to their less concentrated states in the extra-condensate milieu; this ‘condensation’ property enables condensates to organize their functions. Thus, because of high concentrations of specific proteins, we expect condensates to promote protein–protein interactions [e.g., refs. [49,50,51]]. We further narrowed our study of condensate proteins to complexes in which at least one protein is reported to be an autoantigen; we expected that autoantigens would be likely to exhibit interaction-associated changes responsible for new epitopes.

We analyzed nine complexes representing twelve autoantigens with a total of ~30 new or enhanced epitopes. We suggest that this sample size is sufficient to support our hypothesis. Two-thirds of the 30 proposed sites are composed of all or nearly all coil and/or turn, and 80% have either a net positive or negative charge at pH 7. Qiao et al. [52] used computational methods and X-ray structures to analyze the contacts between 350 antigens and their corresponding antibodies; “the presence of charged residues was noticeably large, hydrophobic residues were relatively low,” and turn and coil were highly prevalent (37% and 31%, respectively). Thus, the charge and structural similarity between our 30 epitopes and epitopes in general suggest that our proposed epitopes are a representative sample.

Amino acid residues of some of the proposed new epitopes had secondary structure properties that differed from their secondary structures in the uncomplexed forms. In most cases, the difference was coil-to-turn or vice versa. As indicated, turns are often recognized by antibodies [52,53]. Interestingly, STAT1 residues 130–132 and PES1 residues 455–461 transitioned from helix in the monomer to turn in the complex for which, in both cases, ScanNet predicted new epitopes.

Some of the proposed epitopes did not differ substantially in conformation or secondary structure relative to the same residues in the uncomplexed forms. In addition, some epitopes appeared to have less than 100% accessibility. It is important to note that both AlphaFold 2 Multimer and ScanNet predictions were derived from static structures. Dynamic changes in structure and the nature of ScanNet’s training set could account, in part, for selection of epitope residues that appear unsuitable in static structures. Yet AlphaFold cannot report on protein dynamics nor can it consider features of any condensate milieu in predicting protein structure. And ScanNet’s predictions do not say anything about the degree to which the immune system may react to predicted epitopes.

Dynamic changes in structure may support our finding of potential discontinuous B-cell epitopes in STAT1, ERF1, and BUD23, each of which had two predicted epitopes within 6–7 Å of each other in the tertiary structures. That distance may be sufficiently close proximity as is, or made still closer by dynamic changes in structure, to yield a single discontinuous B-cell epitope [54]. Obviously, additional methods are needed to assess possible collaboration between predicted epitopes.

Note also that AlphaFold returns the five best predicted (static) structures. Individual structures may differ noticeably in places yet contribute only insignificant differences in overall AlphaFold quality metrics. We analyzed only the top-most structure as defined by the quality metrics, despite the possibility that one or more of the other four structures could have led to slightly different ScanNet predictions.

### 4.1. Novelty and Significance of the Study

The process we describe for discerning potential epitopes is simple and effective. Importantly, tools such as AlphaFold and ScanNet enable consideration of proteins as multimeric complexes. Still, our study is only a first step and not a substitute for direct biochemical confirmation of epitopes that arise from protein–protein interaction. The key point of our model is that condensates provide environments in which protein complexes can form efficiently and, in so doing, condensates are proposed to be efficient at generating new epitopes.

As summarized below, the possible participation of biomolecular condensates in the formation of autoantigens leads us to propose several consequences unique to a condensate-mediated mechanism.

### 4.2. Source for Many Autoantigens

The CD-CODE database contains ~4300 human proteins reported to be associated with one or more condensates. Thus, according to our model, there is a large potential for autoantigens to arise within condensates. Indeed, we estimate that the fraction of autoantigens in the human condensate proteome is more than a factor of two greater than the fraction of autoantigens in the entire human proteome (Chi squared *p* < 0.0001; see Section 2). The difference is noteworthy given the large number of autoantigens involved (~950 in condensates), suggesting that protein conformational change may be a significant mechanism of autoantigenicity. Interestingly, healthy people possess IgG autoantibodies—there are upwards of hundreds of autoantibodies in individuals [3,20,55,56]. Perhaps these autoantibodies are a consequence of the contribution of condensates to the population of autoantigens during an individual’s lifetime.

### 4.3. Autoantigenicity Based on Normal Functioning Immune System

A key feature of the condensate-mediated mechanism for autoantigenicity is the absence of any defect in the immune system. An absence of immune dysfunction in autoimmunity is echoed by Matzinger [57], who reminds us that “Many researchers consider autoimmune disease to be due to defects in the immune system, for example, failure of self-tolerance or regulation.” Instead, Matzinger proposes that autoimmunity arises from “a defect in cell physiology, cell death, cell scavenging, or cell processing” that normally prevents an immune response. Similarly, Mustelin and Andrade [58] argue that autoimmunity does not arise from “loss of tolerance” by the immune system but instead from the same types of neoantigens that occur in cancer. Matzinger, Mustelin, and Andrade invoke a normal immune system acting on a defective or mutational process. However, the protein conformational change that presumably occurs in condensates is a by-product of the normal functions of condensates in gene expression, cellular physiology, and cell survival. The possible subsequent appearance of autoantigens arising in condensates may occur because B cells with subtle changes in antigen recognition are constantly being created.

### 4.4. Relation of Model to Immune Tolerance

Our model for the function of condensates in promoting antigenicity should be considered in the context of immune tolerance. Cells use many mechanisms to regulate the assembly and disassembly of condensates [59], often in response to stress. Thus, the emergence of a heretofore absent condensate after the developmental stage, when self-protein tolerization occurs, could result in the appearance of untolerized condensate protein complexes. Even constitutive, always-present condensates could acquire untolerized protein complexes. Condensates are highly dynamic, and their compositions are regulated by many mechanisms, e.g., post-translational modification and control of the levels of key proteins that initiate phase separation [60]. Thus, we suggest that complex-specific epitopes associated with conformational change such as we describe may arise, untolerized, at any time in the life of a cell or individual.

### 4.5. Condensates as a Route to Immune System Discovery

Any proposed mechanism of autoantigenicity must eventually address the question of how intracellular proteins are discovered by B cells. Defects in clearance of apoptotic cells and their released debris is usually invoked to explain immune system access [61]. However, condensates may be part of a pipeline that deliberately releases intracellular proteins. Liu et al. [62] reported finding 18.4% (23/125) of P-body [condensate] proteins in exosomes. On the basis of other experiments, the authors suggest that “there may be a role for P-bodies in the concentrative capture of proteins destined for secretion in EVs [exosomes]”. Han et al. [63] similarly proposed a “relay system” in which proteins first enter condensates, which then pass the proteins to exosomes to be released from cells. As Han et al. say, because exosomes “exchange functional content between cells,” immune cells may be recipients of exosomes bearing condensate protein complexes. In fact, exosomes are reported to modulate several aspects of immune cell activity [64].

### 4.6. Pharmacological Intervention

There are dozens of condensates differing in composition and function. A voluminous literature and the founding of several “condensate” pharmaceutical concerns attest to the fact that condensates are “dysfunctional in many disease states” and attractive targets for condensate-modifying drugs [63,65,66]. In terms of our model of autoantigenicity, as implied earlier, condensates are not defective, but nevertheless there may be a prevention or therapeutic avenue to curbing autoantigenicity. For example, an individual with a family history of developing an autoantibody to a particular protein might receive a condensate-targeting drug that interferes with or lessens the particular protein’s ability to form a conformation-altering complex.

## 5. Conclusions

Our results from computational tools suggest that changes in protein conformation and structure, induced by the formation of protein complexes, gives rise to new or strengthened epitopes in biomolecular condensate proteins. Pending confirmation that these predicted epitopes truly exist in vivo and are recognized by B cells or antibodies, the results would constitute an underappreciated mechanism of autoimmunity, a mechanism that does not occur because of a defective function but instead follows from a normal cellular process. We suggest that this proposed mechanism could be confirmed by screening sera for antibodies that bind protein complexes but which do not bind, or bind less well, to the free forms of the constituent proteins. Because all individuals, healthy or otherwise, possess hundreds of autoreactive antibodies, there is an almost unlimited potential to conduct such an investigation.

## Figures and Tables

**Table 1:** Sources of autoantigens considered in this study.

Study Title	Ref.	Number Autoantigens	Criteria for Identification of Autoantigens
Natural IgG Autoantibodies Are Abundant and Ubiquitous in Human Sera, and Their Number Is Influenced By Age, Gender, and Disease	[21]	715	Human protein microarrays probed with serum samples followed by AlexaFluor-conjugated anti-human IgG. Signals compared with negative controls.
A master autoantigen-ome links alternative splicing, female predilection, and COVID-19 to autoimmune diseases	[22]	745	Autoantigens isolated by “peculiar affinity” for dermatan sulfate. MS identification. About 400 of the proteins confirmed by literature. “Most unconfirmed proteins are structurally similar or share epitopes, so they are likely autoantigens.”
Novel Autoantibodies Related to Cell Death and DNA Repair Pathways in Systemic Lupus Erythematosus	[23]	346	Protein microarray to “identify autoantibodies significantly elevated in SLE patients.” ELISA used to validate 16 upregulated autoantibodies.
Single nucleotide polymorphisms as a prerequisite for autoantigens	[24]	341	Searched Medline and other public databases for autoantigens.
Autoantibodies targeting TLR and SMAD pathways define new subgroups in systemic lupus erythematosus	[25]	120	Protein microarray probed with sera from SLE individuals. ELISA used to confirm selected proteins.
Identification of Novel Native Autoantigens in Rheumatoid Arthritis	[26]	100	Protein microarrays probed with pooled plasma. Twenty-three of 102 proteins confirmed by literature citations.
Total		2367	
Total No duplicates		1925	

**Table 2:** XRCC6 residues proposed as new epitopes in XRCC6-XRCC5 complex.

Amino Acid Residue	Amino Acid	2° Structure ^a^ Monomer	Binding Probability Monomer (×100)	2° Structure ^a^ Complex	Binding Probability Complex (×100)	*R* ^b^
554	R	C	42.1	T	53.6	1.27
555	P	C	33.8	C	52.9	1.57
556	K	C	38.3	C	43.3	1.13
557	V	C	39.4	C	58.7	1.49
558	E	C	31.6	C	41.6	1.32
559	Y	C	38.0	C	56.4	1.48
						
563	E	H	25.6	H	37.9	1.48
						
576	F	C	19.2	C	36.7	1.91
577	T	C	41.1	C	45.9	1.12
						
579	P	H	33.6	H	43.0	1.28
580	M	H	38.6	H	65.7	1.70
						
583	E	H	41.5	H	67.6	1.63
584	A	H	8.3	H	47.7	5.75
						
586	R	H	29.6	H	39.8	1.34
587	A	H	24.5	H	45.0	1.84

^a^ C = coil, T = turn, H = α-helix; ^b^ *R* = Binding probability in Complex ÷ Binding probability in Monomer.

**Table 3:** XRCC5 residues proposed as new epitopes in XRCC6-XRCC5 complex.

Amino Acid Residue	Amino Acid	2° Structure ^a^ Monomer	Binding Probability Monomer (×100)	2° Structure ^a^ Complex	Binding Probability Complex (×100)	*R* ^b^
171	K	T	28.8	T	40.3	1.40
172	E	T	19.3	C	51.3	2.66
173	D	T	25.9	C	59.6	2.30
174	G	T	22.7	C	39.9	1.76
175	S	C	19.2	C	42.8	2.23
176	G	C	21.9	C	40.0	1.83
177	D	C	17.6	C	31.9	1.81
						
242	R	C	14.8	C	38.2	2.58
243	H	C	33.3	C	62.8	1.89
244	S	C	50.2	C	58.6	1.17
245	I	C	44.6	C	63.3	1.42
246	H	C	60.6	E	70.0	1.16
247	W	E	41.4	E	43.3	1.05
						
725	V	C	21.6	T	52.7	2.44
726	D	C	25.8	T	36.6	1.42
727	D	C	26.4	T	30.6	1.16
728	L	C	30.4	T	57.1	1.88
729	L	C	40.1	T	59.2	1.48

^a^ C = coil, T = turn, E = β-strand. ^b^ *R* = Binding probability in Complex ÷ Binding probability in Monomer.

**Table 4:** PES1 residues proposed as new epitopes in PeBoW complex.

Amino Acid Residue	Amino Acid	2° Structure ^a^ Monomer	Binding Probability Monomer (×100)	2° Structure ^a^ Complex	Binding Probability Complex (×100)	*R* ^b^
60	A	C	25.6	C	30.9	1.21
61	A	C	22.1	C	50.0	2.26
62	R	C	22.6	C	37.6	1.66
63	T	C	26.3	C	32.5	1.24
						
108	K	H	55.0	H	60.4	1.10
109	D	H	24.9	H	40.8	1.64
110	N	H	16.0	C	41.2	2.58
111	K	C	37.2	C	42.5	1.14
						
455	N	H	12.6	T	47.5	3.77
456	E	H	9.4	T	33.4	3.55
457	S	H	27.2	T	58.9	2.17
458	E	H	17.2	T	63.8	3.71
459	E	H	10.9	T	38.4	3.52
460	E	H	14.3	T	33.2	2.32
461	E	H	20.2	T	31.3	1.55

^a^ C = coil, T = turn, H = α-helix. ^b^ *R* = Binding probability in Complex ÷ Binding probability in Monomer.

**Table 5:** STAT1 residues proposed as new epitopes in STAT1-STAT3 complex.

Amino Acid Residue	Amino Acid	2° Structure ^a^ Monomer	Binding Probability Monomer (×100)	2° Structure ^a^ Complex	Binding Probability Complex (×100)	*R* ^b^
128	G	T	33.5	T	37.5	1.13
129	N	T	34	C	39.0	1.15
130	I	H	41.2	T	35.0	0.85
131	Q	H	38.5	T	42.6	1.11
132	S	H	20.5	T	41.6	2.03
						
620	S	T	26.4	T	53.4	2.02
621	Q	T	31.4	T	63.2	2.01
622	N	T	30.7	T	46.2	1.50
						
655	A	T	18.8	T	30.7	1.63
656	A	T	29.4	T	46.6	1.59
657	E	T	32.4	T	48.4	1.49
						
711	E	C	46.8	E	61.4	1.31
712	V	C	33.2	C	50.2	1.51
713	H	C	44.6	T	52.1	1.17

^a^ C = coil, T = turn, H = α-helix, E = β-strand. ^b^ *R* = Binding probability in Complex ÷ Binding probability in Monomer.

**Table 6:** Proposed epitopes of ERF1.

Amino Acid Residue	Amino Acid	2° Structure ^a^ Monomer	Binding Probability Monomer (×100)	2° Structure ^a^ Complex	Binding Probability Complex (×100)	*R* ^b^
314	M	H	32.7	H	42.9	1.31
315	G	C	18.5	C	29.9	1.62
316	A	C	24.6	C	33.5	1.36
						
369	I	E	23.1	E	31.5	1.36
370	E	E	29.8	E	41.0	1.38
371	S	E	22.3	E	34.6	1.55
						
414	R	T	34.7	T	43.9	1.27
415	Y	T	46.7	T	57.9	1.24
416	R	T	41.6	T	32.8	0.79
417	V	C	36.4	C	44.1	1.21

^a^ C = coil, T = turn, H = α-helix, E = β-strand. ^b^ *R* = Binding probability in Complex ÷ Binding probability in Monomer.

**Table 7:** Proposed epitope for SSRP1.

Amino Acid Residue	Amino Acid	2° Structure ^a^ Monomer	Binding Probability Monomer (×100)	2° Structure ^a^ Complex	Binding Probability Complex (×100)	*R* ^b^
437	S	C	51.2	T	58.5	1.14
438	Y	C	55.8	T	67.5	1.21
439	D	C	45.3	T	57.9	1.28
440	E	C	44.9	C	62.1	1.38
441	Y	C	44.6	C	61.6	1.38
442	A	C	27.8	C	69.6	2.50
443	D	C	22.8	C	58.0	2.54
444	S	C	23.1	C	59.7	2.58
445	D	C	15.6	T	33.3	2.13
446	E	C	17.0	T	39.3	2.31

^a^ C = coil, T = turn. ^b^ *R* = Binding probability in Complex ÷ Binding probability in Monomer.

**Table 8:** Proposed epitopes for SPT16. Note: Five additional potential epitopes were identified for SPT16; however, they are not shown for the sake of brevity.

Amino Acid Residue	Amino Acid	2° Structure ^a^ Monomer	Binding Probability Monomer (×100)	2° Structure ^a^ Complex	Binding Probability Complex (×100)	*R* ^b^
432	L	C	41.5	C	48.1	1.16
433	K	C	31.8	C	35.7	1.12
434	N	C	21.9	C	33.9	1.55
435	E	G	27.5	C	38.3	1.39
						
846	E	E	14.2	E	37.7	2.65
847	R	T	58.3	T	75.4	1.29
848	V	T	44.8	T	65.3	1.46
849	Q	T	67.3	T	77.3	1.15
850	F	T	77.2	T	79.3	1.03
851	H	T	51.5	T	61.3	1.19
852	L	T	40.6	T	54.6	1.34
853	K	C	29.2	C	36.3	1.24

^a^ C = coil, T = turn, E = β-strand, G = 3_10_ helix. ^b^ *R* = Binding probability in Complex ÷ Binding probability in Monomer.

**Table 9:** Proposed epitopes for BUD23.

Amino Acid Residue	Amino Acid	2° Structure ^a^ Monomer	Binding Probability Monomer (×100)	2° Structure ^a^ Complex	Binding Probability Complex (×100)	*R* ^b^
11	G	C	45.1	C	53.6	1.19
12	G	C	30.9	C	47.0	1.52
13	P	C	33.8	C	51.7	1.53
						
15	E	H	28.0	H	34.7	1.24
16	L	H	31.2	H	40.9	1.31
						
130	N	C	26.0	C	33.5	1.29
131	A	C	26.6	C	33.6	1.26
132	N	C	37.2	C	45.2	1.22
133	K	T	37.8	T	43.5	1.15

^a^ C = coil, T = turn, H = α-helix, E = β-strand. ^b^ *R* = Binding probability in Complex ÷ Binding probability in Monomer.

## Data Availability

AlphaFold data for monomeric structures are available for academic and commercial use, under a CC-BY-4.0 license [74]. Use of Complex Portal data is covered by a Creative Commons Public Domain (CC0) License. CD-CODE is licensed under a Creative Commons Attribution-Share Alike 4.0 International License. Immune Epitope Database data are licensed under a Creative Commons Attribution 4.0 International License. The original contributions presented in this study are included in the article/Supplementary Materials. Further inquiries can be directed to the corresponding authors.

## Supplementary Materials

The following supporting information can be downloaded at: https://www.mdpi.com/article/10.3390/biom16060803/s1, Supplementary File S1: Details of Molecular Dynamics Simulation; Supplementary File S2: Additional Figures; Figure S1: Overlay of portions of XRCC5 monomer (sand color) and in complex (cyan) with XRCC6 (XRCC6 not shown for clarity); Figure S2: Additional proposed epitopes in PES1; Figure S3: STAT1 epitope residues 128–132. STAT1 monomer is in sand color; Figure S4: Proposed new epitope, residues 369–371, of ERF1 in the ERF1-GSPT1 complex; Supplementary File S3: Additional Complexes; Figure S5: Proposed new epitope in HSP90-CDC37 complex. Shown in sand color is a portion of the structure of free HSP90 predicted by AlphaFold2 Multimer; Figure S6: Proposed new KPNA2 (importin-α) epitopes; Figure S7: Proposed new KPNB1 epitopes; Figure S8: Proposed new epitope in EIF2S1; Table S1: Proposed new epitope for HSP90 in complex with CDC37; Table S2: Proposed new KPNA2 epitopes in KPNA2-KPNB1 heterodimer; Table S3: Proposed new KPNB1 epitopes in KPNA2-KPNB1 heterodimer; Table S4: Proposed epitope for EIF2S1; Supplementary File S4: Epitope Summary Table. References [67,68,69,70,71,72,73] are cited in the Supplementary Materials.
