# Supplementary material for: A Novel Mechanism for Autoantigenicity: Condensate Conformational Change
Source: Biomolecules. 2026 May 29;16(6):803. doi: 10.3390/biom16060803 (PMC13296523; doi:10.3390/biom16060803)
Supplement: Supplementary file 1 [file biomolecules-16-00803-s001.zip › biomolecules-4236916 Supplementary File S1-Molecular Dynamics.pdf]

biomolecules

Supplementary File S1, Molecular Dynamics Simulation

Experimental details

A molecular dynamics (MD) simulation of the XRCC6-XRCC5 protein complex was performed using the GROMACS software (version 2024.4) on the Neurosnap computational platform <https://neurosnap.ai/>. The structure was parameterized using the AMBER99SB-ILDN force field. The protein-ligand system was placed within a cubic solvent box with a minimum distance of 0.8 nm between the protein and box edges and solvated using TIP3P water molecules. Ion concentrations were set to 0.15 M NaCl, maintaining system neutrality with sodium (Na^+) and chloride (Cl^-) ions.

Energy minimization was conducted using the steepest descent algorithm until convergence to a maximum force below 500 kJ/mol/nm. The minimization step size was set to 0.01 nm, with energy outputs recorded every 500 steps. Nonbonded interactions were managed with a short-range electrostatic cutoff of 1.0 nm and a van der Waals cutoff of 1.0 nm using the Potential-shift-Verlet modification. Long-range electrostatics were treated with the Particle Mesh Ewald (PME) method, and dispersion corrections were applied for energy and pressure.

Following energy minimization, equilibration was performed in two phases. The first phase employed an NVT (constant Number, Volume, Temperature) ensemble at 300 K for 100 ps using the velocity-rescaling thermostat with a time constant of 1.0 ps. All bonds were constrained using the LINCS algorithm with an order of 8 and four iterations. Initial velocities were assigned from a Maxwell distribution at 300 K. The second phase involved an NPT (constant Number, Pressure, Temperature) ensemble for an additional 100 ps at 1 bar pressure, using the C-rescale barostat with isotropic scaling, a time constant of 5.0 ps, and an isothermal compressibility of 4.5×10^{-5} bar^{-1}. The same thermostat and nonbonded interaction parameters from the NVT phase were applied.

Following equilibration, the system was subjected to a 100 ns production MD run at 300 K and 1 bar pressure using the leapfrog integrator with a 1 fs timestep. Periodic boundary conditions were applied in all three dimensions, and long-range electrostatic interactions were calculated using the PME method with a cutoff of 1.0 nm. Lennard-Jones interactions were truncated at 1.0 nm with a Potential-shift-Verlet modification. Pressure coupling was performed using the C-rescale algorithm with isotropic box scaling and a time constant of 5.0 ps. The velocity-rescale thermostat was applied with a coupling time of 1.0 ps. Hydrogen bond constraints were enforced using the LINCS algorithm with an order of 8 and four iterations. Dispersion corrections for energy and pressure were applied to account for cutoff truncation effects.

Results from the simulation, including detailed trajectory and analysis data, are freely accessible from the Neurosnap platform at https://neurosnap.ai/job/6791957b99464b271efcaf1d?share=67a535fecb419ef7b4982b42

Results

The molecular dynamics (MD) simulation of the XRCC6-XRCC5 protein complex revealed key indicators of structural stability and favorable complex formation. Backbone and alpha-carbon root-mean-square deviation (RMSD) analysis showed an initial period of fluctuation that stabilized after ~4.35 ns, indicating that the complex reached a conformational equilibrium early in the simulation. This conformational equilibrium suggested

that large-scale structural rearrangements were minimal, supporting the conclusion that the complex remained stable under simulation conditions.

The radius of gyration (Rg) steadily decreased over time before reaching a plateau, signifying a progressive compaction of the XRCC6-XRCC5 complex. The absence of major fluctuations in Rg after stabilization further reinforced the formation of a well-packed and energetically favorable protein-protein interface.

Hydrogen bond analysis revealed an increasing trend throughout the simulation, albeit with minor fluctuations. These minor fluctuations suggested that inter-protein interactions continued to stabilize over time, further supporting the stable association of XRCC6 and XRCC5. The increasing number of hydrogen bonds indicated favorable molecular interactions contributing to the persistence of the complex.

Root-mean-square fluctuation (RMSF) analysis showed limited flexibility across most of the protein complex, with the exception of two distinct regions: residues 540–609 of XRCC6 and residues 550-585 of XRCC5. The fluctuations in these regions were consistent with expectations, as the affected segments correspond to the XRCC6 C-terminal region and a loop on XRCC5. The localized flexibility of these regions did not appear to affect the overall stability of the complex.
