# Supplementary material for: A Novel Mechanism for Autoantigenicity: Condensate Conformational Change
Source: Biomolecules. 2026 May 29;16(6):803. doi: 10.3390/biom16060803 (PMC13296523; doi:10.3390/biom16060803)
Supplement: Supplementary file 1 [file biomolecules-16-00803-s001.zip › biomolecules-4236916 Supplementary File S2-Additional Figures.pdf]

biomolecules

Supplementary File S2, Additional Figures

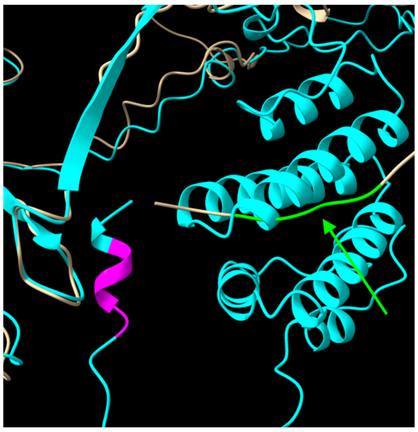

Figure S1. Overlay of portions of XRCC5 monomer (sand color) and in complex (cyan) with XRCC6 (XRCC6 not shown for clarity). Epitope residues 725–729 are green in monomer (green arrow at right) and magenta in the XRCC6-XRCC5 complex.

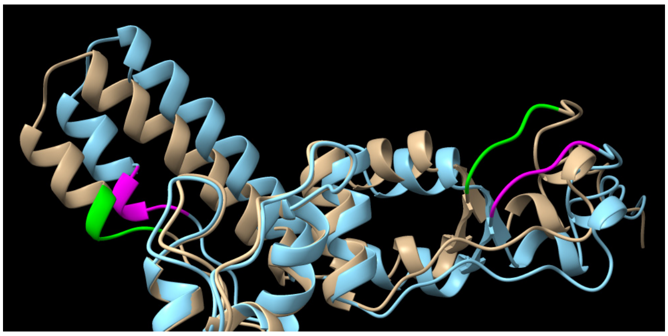

Figure S2. Additional proposed epitopes in PES1. A portion of the PES1 monomer is shown in sand color; the same portion of PES1 in the PeBoW complex is in cyan. Proposed epitope residues 108–111 are at left, green for monomer and magenta for complex. Proposed epitope residues 60–63 are at right, same color scheme. Partner proteins BOP1 and WDR12 not shown.

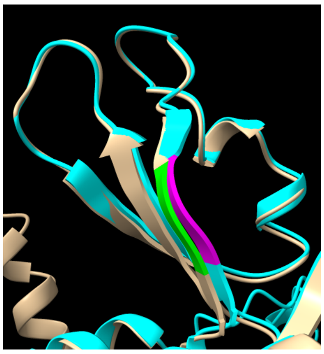

Figure S3. STAT1 epitope residues 128–132. STAT1 monomer is in sand color; STAT1 in complex with STAT3 is cyan (STAT3 not shown). Epitope residues are green in monomer, magenta in complex. Note the complete flip of the helix bundle between monomer and complex, perhaps promoted by the all-turn epitope residues in the complex.

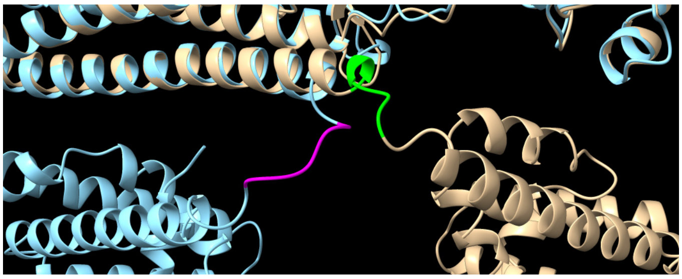

Figure S4. Proposed new epitope, residues 369–371, of ERF1 in the ERF1-GSPT1 complex. Portion of free monomer is in sand color; the same portion in complex is cyan. Green is epitope in monomer, magenta in complex. Partner protein GSPT1 not shown.
