# Supplementary material for: A Novel Mechanism for Autoantigenicity: Condensate Conformational Change
Source: Biomolecules. 2026 May 29;16(6):803. doi: 10.3390/biom16060803 (PMC13296523; doi:10.3390/biom16060803)
Supplement: Supplementary file 1 [file biomolecules-16-00803-s001.zip › biomolecules-4236916 Supplementary File S3-Additional Complexes.pdf]

Supplementary File S3, Additional Complexes

A. HSP90-CDC37

The HSP90-CDC37 (Complex Portal #CPX-3288) is a trimer composed of two HSP90 molecules and one CDC37 molecule. Together, the proteins are a molecular chaperone that activates a great many substrate proteins, principally protein kinases [67].

The pTM and ipTM metrics of the complex were 0.79 and 0.76. Both proteins are autoantigens [22]. For the complex, we found one potential new epitope in HSP90 (Table S1 and Figure S5 below) and no new epitope in CDC37. The new epitope in HSP90 is derived from an 8-residue helix-to-turn transition, an occurrence identical to the 7-residue helix-to-turn transition in PES1 (see Results, section 3.2.2). In both instances, the resulting new epitopes are robust, as shown by the high R values. Note that both HSP90 molecules in the complex showed the 8-residue turn. In addition, the region containing the proposed new epitope is not masked by CDC37 (not shown).

Table S1. Proposed new epitope for HSP90 in complex with CDC37.

Amino Acid Residue	Amino Acid	2° Structure[a] Monomer	Binding probability Monomer (×100)	2° Structure[a] Complex	Binding probability Complex (×100)	*R*[b]
237	E	H	6.0	T	47.8	7.97
238	K	H	7.0	T	50.6	7.22
239	E	H	5.7	T	41.9	7.35
240	D	H	6.2	T	52.9	8.53
241	K	H	8.0	T	56.2	7.03
242	E	H	8.5	T	46.0	5.41
243	E	H	9.3	T	41.4	4.45
244	E	H	8.9	T	34.5	3.88

[a]H= α-helix, T=turn.

[b]*R*=Binding probability in Complex ÷ Binding probability in Monomer.

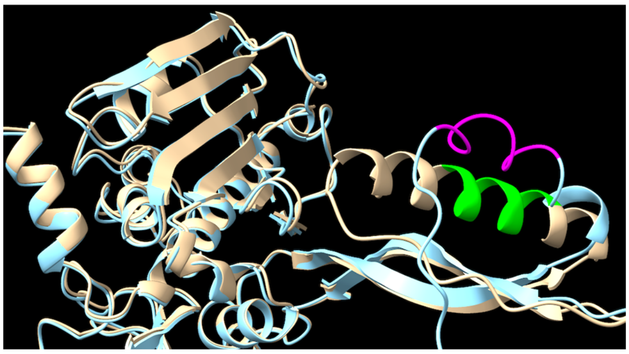

Figure S5. Proposed new epitope in HSP90-CDC37 complex. Shown in sand color is a portion of the structure of free HSP90 predicted by AlphaFold2 Multimer. The structure of one HSP90 chain in the complex is overlaid in cyan (CDC37 not shown). The proposed new epitope in HSP90 is shown in magenta, and green indicates the same residues in the free HSP90 monomer.

B. KPNA2-KPNB1 nuclear transport complex

The KPNA2-KPNB1 (aka importin-α/β and karyopherin-α/β) heterodimer (Complex Portal #CPX-1027) ferries proteins from the cytoplasm into the nucleus [46]. Both subunits are autoantigens [21,22,68]. CD-CODE indicates that both proteins are associated with the nucleolus and the Stress Granule. KPNA2 and KPNB1 are unlikely to form a complex in the nucleolus because RanGTP, which dissociates KPNA2 from KPNB1, is present at high concentration in the nucleus. Conversely, RanGTP is essentially absent from the cytoplasm; thus, the two proteins could interact in the Stress Granule. The AlphaFold Multimer metrics of the predicted complex were pTM=0.63, ipTM=0.81.

KPNA2

Of the 529 amino acids in KPNA2, 119 (22%) showed a >2 Å conformational difference between monomer and complex. Table S2 lists two groups of residues that met the criteria for new or enhanced epitopes in the KPNA2-KPNB1 heterodimer, namely residues 22–27 and 58–68. Note: Data for residues 24, 59, and 61 are not shown because those residues did not meet the ScanNet criteria for B-cell binding. Instead, 24, 59, and 61 may be examples of "indifferent residues" or "non-contact residues" [41,69] common in B-cell epitopes; such residues are not involved in interaction with antibodies (and, by inference, the B-cell receptor), but they may be important in maintaining the overall structures of epitopes.

Table S2. Proposed new KPNA2 epitopes in KPNA2-KPNB1 heterodimer.

Amino Acid Residue	Amino Acid	2° Structure[a] Monomer	Binding probability Monomer (×100)	2° Structure[a] Complex	Binding probability Complex (×100)	R^b
22	K	C	26.8	T	41.4	1.54
23	D	C	13.3	T	33.1	2.49
25	T	H	29.0	H	37.9	1.31
26	E	H	15.5	H	44.4	2.86
27	M	H	13.1	H	38.1	2.91
58	D	T	23.8	T	40.5	1.70
60	A	T	21.6	C	42.5	1.97
62	S	T	5.7	C	42.6	7.47
63	P	T	22.5	C	41.9	1.86
64	L	T	10.1	C	35.6	3.52
65	Q	T	14.5	C	46.6	3.21
66	E	C	11.6	C	33.1	2.85
67	N	T	11.8	C	45.7	3.87
68	R	T	20.7	C	33.5	1.62

[a]C=coil, T=turn, H=α-helix.

[b]*R*=Binding probability in Complex ÷ Binding probability in Monomer.

Heterodimerization of KPNA2 and KPNB1 occurs by interaction between residues 11–54 of KPNA2, known as the importin beta-binding (IBB) domain [70], and KPNB1. The IBB domain is composed of two α-helices, residues 11-23 and 24-51. In the AlphaFold2-predicted monomer, the entire IBB domain is positioned away from the main body of KPNA2

and accessible (Figure S6A). In the complex, the IBB domain is completely surrounded by KPNB1 and located in a central cavity of KPNB1 (not shown; see [71] for details). Thus, it is not clear why residues 22–27 of the IBB domain are predicted by ScanNet to have greater B-cell binding probability in the complex. Nevertheless, the probabilities for IBB residues 22, 23, 25, 26, and 27 are lower by 20–30% for the assembled complex compared with the individual chain, consistent with some degree of inaccessibility.

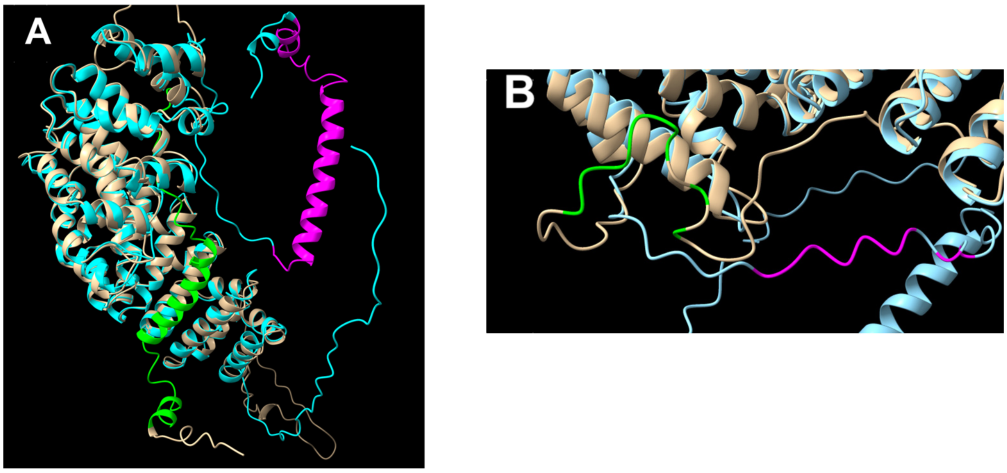

Figure S6. Proposed new KPNA2 (importin-α) epitopes. (A) Overlay of KPNA2 monomer (sand) and KPNA2 in complex (cyan) with KPNB1 (KPNB1 not shown for clarity). Green and magenta show, respectively, IBB domain in monomer and in complex. (B) Additional proposed new KPNA2 epitope in KPNA2-KPNB1 complex. Green and magenta show, respectively, residues 58–68 in monomer and complex. Binding partner KPNB1 not shown.

Residues 58–68 are part of a large loop-like structure composed of nearly all turn residues (Table S2, Figure S6B). The loop has become mostly unstructured (nearly all coil) in the KPNA2-KPNB1 complex, possibly residing in a large cavity bounded at the bottom by the IBB helix. This suggestion is consistent with the fact that the binding site probabilities for KPNA2 residues 58–68 in the assembled complex were 40–50% lower compared with the probabilities for the individual KPNA2 chain in the complex (data not shown). Our rule was to disregard large unstructured segments as acceptable epitopes; yet, the binding probabilities ratios for 58–68 were among the highest we observed, suggesting that the all-T to all-C transition is significant with respect to generating a new epitope.

KPNB1

Of the 876 residues in KPNB1, 263 (30%) had >2 Å difference in conformation between monomer and complex forms. Two blocks of residues are suggested to be part of new KPNB1 epitopes in the KPNA2-KPNB1 heterodimer (Table S3). Residues 659–663 have identical structures and nearly identical locations (Figure S7). As well, 659–663 have essentially identical binding site probabilities in the assembled and individual chain (data not shown). Residues 839-841 appear more accessible compared with 659–663, but equally so in monomer and complex (Figure S7) as suggested by the binding values for the assembled and individual KPNB1 chains (not shown).

Table S3. Proposed new KPNB1 epitopes in KPNA2-KPNB1 heterodimer.

Amino Acid Residue	Amino Acid	2° Structure[a] Monomer	Binding probability Monomer (×100)	2° Structure[a] Complex	Binding probability Complex (×100)	R^b
659	K	C	24.6	C	46.1	1.87
660	N	T	39.9	T	63.1	1.58
661	Y	T	28.3	T	43.6	1.54
662	A	T	42.6	T	70.7	1.66
663	E	T	47.7	T	65.6	1.38
839	A	T	20.3	C	34.0	1.67
840	R	T	29.3	C	43.0	1.47
841	P	H	37.9	H	53.3	1.41

[a]C=coil, T=turn, H=α-helix.

[b]R=Binding probability in Complex ÷ Binding probability in Monomer.

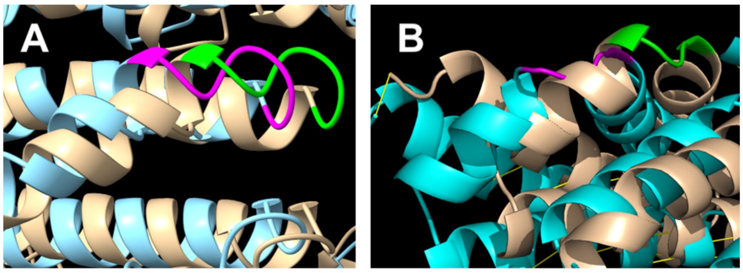

Figure S7. Proposed new KPNB1 epitopes. (A) Residues 659–663. (B) Residues 839–841. KPNB1 monomer is sand color; epitope residues in monomer are green (top). KPNB1 in complex is cyan. Epitope residues are magenta. Binding partner KPNA2 not shown.

C. *EIF2* translation initiation factor complex

The eukaryotic translation initiation factor 2 complex (EIF2), a heterotrimer composed of EIF2S1α, EIF2S2β, and EIF2S3γ (Complex Portal #CPX-2716), brings the Met-tRNA^{Met} to the 40S ribosomal subunit in the translation initiation process [72]. All three proteins are in the Stress granule and nucleolus. EIF2S1α is the autoantigen [22,73]. The AlphaFold Multimer structure metrics for the predicted complex were pTM=0.62 and ipTM=0.65.

Most of the EIF2S1 residues (84%) had >2 Å differences in conformation between monomer and complex forms. Despite the substantial number of differences, only one triplet, residues 66–68, was predicted as a new epitope (Table S4). The B cell binding probabilities of these residues in the assembled complex were the same as for the individual EIF2S1 chain in the complex (not shown), indicating that the three residues were completely accessible in the complex. The triplets are ~5 Å apart comparing monomer with complex (Figure S8).

Table S4. Proposed epitope for EIF2S1.

Amino Acid Residue	Amino Acid	2° Structure[a] Monomer	Binding probability Monomer (×100)	2° Structure[a] Complex	Binding probability Complex (×100)	R^B
66	G	T	50.3	T	58.3	1.16
67	R	E	41.2	E	50.0	1.21
68	N	E	37.2	E	50.8	1.37

[a]T=turn, E=β-strand.

[b]*R*=Binding probability in Complex ÷ Binding probability in Monomer.

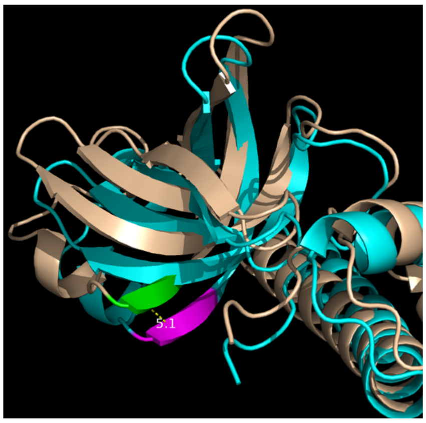

Figure S8. (Above, Left). Proposed new epitope in EIF2S1. The monomer (sand) is overlaid with the complex form of EIF2S1 (cyan). Green shows residues 66–68 in the monomer; magenta shows the residues in the complex. The two triplets are ~5.1 Å apart. The other two proteins in the complex, EIF2S2 and EIFS3, are not shown.
