# Supplementary material for: A Novel Mechanism for Autoantigenicity: Condensate Conformational Change
Source: Biomolecules. 2026 May 29;16(6):803. doi: 10.3390/biom16060803 (PMC13296523; doi:10.3390/biom16060803)
Supplement: Supplementary file 1 [file biomolecules-16-00803-s001.zip › biomolecules-4236916 Supplementary File S4-Summary Table.pdf]

biomolecules

MDPI

Supplementary File S4

Epitopes from protein-protein interaction associated with biomolecular condensates and described in accompanying report.

Protein UniProt ID	Protein Name	Partner(s)	Epitope(s)	Location (aa residues)	Predominant Structure	New or Enhanced Epitope[1]
P12956	XRCC6	XRCC5	RPKVEY-E-FT-PM-EA-RA[2]	554–559, 563, 576–577, 579–580, 583–584, 586–587	Coil+Helix	Enhanced (coil) New (helix)
P13010	XRCC5	XRCC6	KEDGSGD	171–177	Coil	New
			RHSIHW	242-247	Coil	Enhanced
			VDDLL	725–729	Turn	New
O00541	PES1	BOP1+WDR12	AART	60–63	Coil	New
			KDNK	108–111	Helix	New
			NESEEEE	455–461	Helix	New
P42224	STAT1	STAT3	GNIQS	128–132	Turn	Enhanced
			SQN	620–622	Turn	New
			AAE	655–657	Turn	New
			EVH	711–713	Turn	Enhanced
P62495	ERF1	GSPT1	MGA	314–316	Coil	New
			IES	369–371	β-strand	New
			RYRV	414–417	Turn	Enhanced
Q08945	SSRP1	SPT16	SYDEYADSDE	437–446	Coil+Turn	New+Enhanced
Q9Y5B9	SPT16[3]	SSRP1	LKNE	432–434	Coil	New
O43709	BUD23	TRMT112	ERVQFHLK	846–853	Turn	Enhanced
P52292	KPNA2	KPNB1	KD-TEM	22, 23, 25–27	Turn+Helix	New
			D-A-SPLQENR	58, 60, 62–68	Coil	New
Q14974	KPNB1	KPNA2	KNYAE	659–663	Turn	New
			ARP	839–841	Coil	New
P05198	EIF2S1α	EIF2S1β+EIF2S1γ	GRN	66–68	β-strand	Enhanced
P07900	HSP90	CDC37	EKEDKEEE	237–244	Turn	New

[1]New indicates that the B-cell binding site probability is all or mostly < 35% in the monomer and mostly > 35% in the complex. Enhanced indicates that the B-cell binding site probability is all or mostly > 35% in the monomer but all or most values are yet greater for the complex. See Main text Results section.

[2]It is unknown whether the XRCC6 epitope is a single, interrupted segment or whether there are several separate epitopes. For the purpose of this compilation, all the indicated residues are considered as a single, interrupted epitope.

[3]Five additional proposed epitopes were identified for SPT16; they are not shown for the sake of brevity.
